# Playing with Low Amounts of Expanded Graphite for Melt-Processed Polyamide and Copolyester Nanocomposites to Achieve Control of Mechanical, Tribological, Thermal and Dielectric Properties

**DOI:** 10.3390/nano14070606

**Published:** 2024-03-29

**Authors:** Ruben Vande Ryse, Michiel Van Osta, Mounia Gruyaert, Maarten Oosterlinck, Ádám Kalácska, Mariya Edeleva, Frederik Pille, Dagmar R. D’hooge, Ludwig Cardon, Patrick De Baets

**Affiliations:** 1 Centre for Polymer and Material Technologies (CPMT), Department of Materials, Textiles and Chemical Engineering, Ghent University, Technologiepark-Zwijnaarde 130, 9052 Ghent, Belgium; ruben.vanderyse@ugent.be; 2 Internet Technology and Data Science Lab (IDLab), Department of Information Technology (INTEC), Ghent University—imec, Technologiepark-Zwijnaarde 126, 9052 Ghent, Belgium; michiel.vanosta@imec.be; 3 Department of Large Animal Surgery, Anaesthesia and Orthopaedics, Faculty of Veterinary Medicine, Ghent University, Salisburylaan 133, 9820 Merelbeke, Belgium; mounia.gruyaert@ugent.be (M.G.); frederik.pille@ugent.be (F.P.); 4 Soete Laboratory, Department of Electromechanical, Systems and Metal Engineering, Ghent University, Technologiepark-Zwijnaarde 46, 9052 Ghent, Belgium; adam.kalacska@ugent.be (Á.K.); patrick.debaets@ugent.be (P.D.B.); 5 Laboratory for Chemical Technology (LCT), Department of Materials, Textiles and Chemical Engineering, Ghent University, Technologiepark-Zwijnaarde 125, 9052 Ghent, Belgium; dagmar.dhooge@ugent.be; 6 Centre for Textiles Science and Engineering (CTSE), Department of Materials, Textiles and Chemical Engineering, Ghent University, Technologiepark-Zwijnaarde 70A, 9052 Ghent, Belgium; 7 Flanders Make @ UGent-Core Lab MIRO, 9000 Ghent, Belgium; 8 Systems and Component Design, School of Electrical Engineering and Computer Science, Royal Institute of Technology KTH, Lindstedtvägen 3, 100 44 Stockholm, Sweden

**Keywords:** polymer composites, expanded graphite, dielectric properties, tribology, material characterisation

## Abstract

Polyamide 11 (PA11) and copolyester (TPC-E) were compounded through melt extrusion with low levels (below 10%) of expanded graphite (EG), aiming at the manufacturing of a thermally and electrically conductive composite resistant to friction and with acceptable mechanical properties. Thermal characterisation showed that the EG presence had no influence on the onset degradation temperature or melting temperature. While the specific density of the produced composite materials increased linearly with increasing levels of EG, the tensile modulus and flexural modulus showed a significant increase already at the introduction of 1 wt% EG. However, the elongation at break decreased significantly for higher loadings, which is typical for composite materials. We observed the increase in the dielectric and thermal conductivity, and the dissipated power displayed a much larger increase where high frequencies (e.g., 10 GHz) were taken into account. The tribological results showed significant changes at 4 wt% for the PA11 composite and 6 wt% for the TPC-E composite. Morphological analysis of the wear surfaces indicated that the main wear mechanism changed from abrasive wear to adhesive wear, which contributes to the enhanced wear resistance of the developed materials. Overall, we manufactured new composite materials with enhanced dielectric properties and superior wear resistance while maintaining good processability, specifically upon using 4–6 wt% of EG.

## 1. Introduction

Polymer materials find a wide range of applications because of their specific material properties, e.g., lower stiffness and ease of manufacturing [1]. However, the application of virgin (unmodified) polymers in electronics demanding, for example, electromagnetic interference (EMI) control, electrical conductivity (EC) and thermal conductivity (TC), is limited, since polymer materials are generally regarded as insulators [2,3].

Polymer-based composites [2,4,5] have offered solutions in many cases, including microelectronics, as the properties of the virgin polymer matrix can be altered according to the desired application [6]. Much research has been conducted using different kinds of fibres, fillers and additives including carbon fibres [7,8] or aramid fibres [9], and glass fibres [10]. Fillers previously investigated include organic fillers, e.g., starches, and metal fillers [11], particularly with alumina [12] or beryllium oxide [13] to increase the EC and TC of the polymer-based composite for microelectronic applications. Additives have also been employed to alter the properties of polymer matrix materials, e.g., as lubricants, plasticizers, stabilizers, anti-shock agents, and pigments [6]. However, if the filler content is above 10%, the processability of the polymer-based composite may be changed too much compared to the virgin polymer material, requiring adjustment of the processing conditions and limiting the manufacturing of complex shapes [14]. Consequently, it remains challenging to manufacture a polymer-based composite material with valuable properties for microelectronic applications while keeping its processability within conventional condition limits. 

To alter/increase the EMI, EC, TC or mechanical properties of composites, carbon-based fillers [15] have been of increasing interest due to their high intrinsic EC and TC, stability, light weight and low cost [16,17]. Many allotropic forms of carbon, e.g., graphene, carbon nanotubes (CNTs), graphene oxide (GO) and expanded graphite (EG) [18], have been studied within the scope of material property modifications [19,20]. Graphene is a single sheet of carbon atoms, while GO is prepared by treating graphite flakes with oxidizing agents, introducing polar groups onto the graphite surface [19]. CNTs are commonly referred to as rolled-up graphene sheets which can be further divided into single-walled carbon nanotubes (SWCNTs) or multi-walled carbon nanotubes (MWCNTs), depending on the number of graphene sheets [18]. EG consists of stacks of nanosheets, which form a worm-like accordion which generally shows a good affinity with polymer materials. For the latter reason, the influence of the addition of EG has been investigated by many groups [21,22,23,24,25,26].

The wide range of molecular and morphological properties of the fillers was reviewed by Wieme et al. [2]. These authors considered variations in filler types and shapes, the dispersion quality of the filler, the mixing of matrix and filler material, and the processing method. It is specifically stated that the aspect ratio (AR), i.e., the ratio of the length of the filler to the cross-sectional diameter, is an important property, as fillers with a large AR can form more easily an endlessly interconnected filler network with lower percentages of filler present. This interconnected network acts as a thermal/electrical conductive network within the polymer matrix and leads to a significant increase in EC and TC [27,28], although the EC and TC properties are anisotropic and should be considered and measured as such. The dispersion quality of the filler is of high importance for forming the latter network, as poor dispersion (e.g., aggregation) will lead to gaps in the network and limit the EC/TC potential.

Furthermore, Wieme et al. [2] mentioned that the main methods which can ensure a homogeneous dispersion of the filler are (i) in situ polymerization, (ii) solution compounding and (iii) melt mixing [19], with the latter showing the most relevance for industrial applications [2] due to its wide applicability in industry, cost efficiency and low environmental impact. The production of composites through a conventional melt blending, however, establishes the percolation threshold (PT), i.e., the filler content at which the interconnected network is formed [29,30], only at marginally higher loading levels compared to the other blending techniques, as the dispersion of the filler is determined by the shear stress applied to the material during processing. Hence, it is worthwhile to explore if one can attain the PT at very low filler-loading levels via an optimized melt-mixing processing method, while keeping processing conditions similar to the conditions for the virgin polymer material.

Supported by literature data dealing with already lower and higher amounts of fillers [1,21,23,24,31,32,33,34,35], it seems most interesting to focus on the better implementation of EG into a polymer matrix. Gupta et al. [1], for instance, observed the electrical PT between 1 and 2 wt% EG, within a polycarbonate matrix by solution compounding. Zheng et al. [32] in turn prepared poly(methyl methacrylate) (PMMA)–EG composites through direct solution blending, and found that the PT was already attained at 1 wt% of EG. Zheng et al. [23], in turn, found the PT for the electrical conductivity of high-density polyethylene (HDPE)-EG composites, prepared via melt blending, at 3 wt%, whilst the PT for untreated graphite was only found at 5 wt%. In the latter investigation, the authors also observed an increase in mechanical properties due to the addition of EG to the HDPE matrix. In addition, Goyal et al. [34] investigated the effects of increasing EG content in a polystyrene (PS) matrix on the thermogravimetric analysis (TGA), dynamic mechanical analysis (DMA) and dielectric results by solution blending the composites. The PT for the electrical properties was found at 2.5 vol% EG, although the dielectric constant decreased significantly at higher frequencies. The study by Sever et al. [36], for example, found a PT for the electrical conductivity of HDPE-EG composites around 5 wt%, whilst also noting a more rapid increase in the Young’s modulus *E* in cases where loading levels were above 10 wt%. Simultaneously, significant increases in storage modulus (*E*′) were found for loading levels up to 40 wt%. The study of Debelak et al. [37] reported similar increases for the flexural modulus (*E_flex_*), for EC and TC for filler amounts between 0 and 20 wt%, stating that higher loading levels would become too difficult to process via solution blending. Other studies [38,39] also reported significant increases in *E*′ with the addition of EG to a polymer matrix. Yousefzade et al. [38], for instance, found the PT for EC around 6–8 wt% in an ethylene-vinyl acetate (EVA) matrix processed through melt compounding, while *E*′ increased significantly between 6 and 15 wt%. Zhang et al. [39] found, by melt processing of polyethylene terephthalate (PET) and EG, the PT for EC at around 3 vol%, while significantly more rapid increases for *E*′ were found at 9 vol%.

It should be noted that moving electronic components may be subjected to friction, which affects the wear of the part and limits the lifetime, and thus more than the conventional mechanical properties should be considered [40]. Several studies [41,42,43,44,45] have thus looked into the effect of graphite or CNTs on the tribological properties of the polymer-composites. Kumar et al. [41], particularly, showed a lubricating effect of graphite on the polyamide 6 (PA6) matrix, whilst also showing a decreasing trend in the specific wear rate (*K*) with the addition of graphite. Jia et al. [46] reported that the coefficient of friction (COF) and *K* of polyimide-based composites dropped significantly with the addition of 5 wt% of EG. Jin et al. [47] compared the effect of natural graphite (NG) and EG on frictional properties of composites and noticed a better lubrication performance of EG compared to NG. Furthermore, the *K* of EG composites decreased significantly. Depending on the application area, the decrease in COF might be desired, in combination with controlled TC and EC properties, e.g., in the electronics of devices such as robotic gripper arms.

In the present work, we investigate the possibility of increasing the composite TC and dielectric properties, i.e., the dielectric constant and dissipated power, without significantly altering the other properties such as the mechanical stiffness/flexibility. For that, we aim at the addition of low, i.e., below 10%, amounts of EG to also keep the processability of the material within a typical processing window. We also aim at decreasing the coefficient of friction and reducing the wear of the material. We also investigate the increase of dielectric properties at much higher frequencies than previously reported [27,31,34,48] (up to 10 GHz) to push the limit of the composite material application. As the polymer matrix, we selected PA11, based on the studies of Kumar et al. [41], and thermoplastic co-polyester-elastomer (TPC-E) Arnitel^®^ EL 740 (DSM Engineering Materials, Heerlen, The Netherlands). Both materials are intended for high-performance applications, e.g., electronics and high thermally stable components, and thus the introduction of thermo-conductive properties can be beneficial. Furthermore, both materials are often processed via injection moulding which is the method we selected in this contribution. Furthermore, EG in PA and TPE polymers exhibit favourable interfacial interactions due to their surface free energies: 53.6 mJ·m^−2^, 34 mJ·m^−2^, and ca. 40 mJ·m^−2^, respectively.

We include a detailed overview of the effect of low-weight percentages of the filler on both the tribological, mechanical and thermal properties, combining in a single contribution a wide spectrum of property analyses, including tribological.

## 2. Materials and Methods

### 2.1. Materials

As matrix materials, we used Polyamide 11 (PA11) Rilsan^®^ BMNO by Arkema (Colombes, France) and thermoplastic co-polyester-elastomer (TPC-E) Arnitel^®^ EL 740 by DSM Engineering Materials (Heerlen, The Netherlands). Both materials are injection moulding grades. The technical datasheets state a tensile modulus of 1280 and 800 MPa, respectively. Furthermore, the density values (25 °C) mentioned are 1030 kg∙m^−3^ for PA11 and 1290 kg∙m^−3^ for TPC-E.

The expanded graphite (EG) used was SIGRATHERM GFG75, by SGL Carbon GmbH (Wiesbaden, Germany). The median particle diameter (50th percentile of the cumulative size distribution), as stated in the technical datasheet, is 75 µm and the powder density is 120 kg∙m^−3^.

### 2.2. Manufacturing

The described matrix materials and EG were used to produce composites with 1, 2, 4 and 6 weight percentage (wt%) of filler via melt mixing in a co-rotating twin screw extruder (APV MP19TC-40 Baker, Peterborough, UK). The produced filament was directly cooled in a water bath at room temperature and shredded into pellets for injection moulding (IM). Pellets were dried overnight at 60 °C prior to the injection moulding step. The IM machine used to produce all relevant samples is an Engel ES 330/80 HL Injection Moulding machine (Engel, Schwertberg, Austria).

General IM processing conditions for both matrix materials are shown in Table 1. The same processing parameters are used as the corresponding virgin matrix material. A temperature design, as seen in Table 1, was used for both extrusion and injection moulding of the compounds.

### 2.3. Characterisation

The specific heat capacity (*C_p_*) was measured using differential scanning calorimetry (DSC) on a Netzsch DSC 214 (Selb, Bayern, Germany), according to the ASTM E1269 standard, after drying the specimens overnight at 60 °C. All measurements were performed under a nitrogen (*N*_2_) atmosphere with a flow rate of 40 mL∙min^−1^, over the range of 5 to 60 °C, with a heating rate of 20 °C∙min^−1^. The *C_p_* value at 20 °C was used for the anisotropic TC measurements.

Thermal properties of the produced compounds were analysed via thermogravimetric analysis (TGA) on a Netsch STA 449 F3 (Selb, Bayern, Germany) and by DSC measurements on a Netzsch DSC 214 (Selb, Bayern, Germany), according to the ISO 11 358-1 and ISO 11 357-1, respectively. All measurements were performed at a heating rate (*β*) of 10 °C·min^−1^ under a nitrogen atmosphere with a flow rate of 50 mL·min^−1^ for TGA and 40 mL·min^−1^ for DSC. The temperature interval used for TGA measurements was [50, 600] °C, while a temperature interval of [50, 240] °C was used for DSC measurements.

Microscopy images were taken on a Keyence (Osaka, Japan) VHX-7000 microscope with a VH-Z20R lens. Three-dimensional stitches (5 × 5) were taken of the pin samples after tribological tests with the Keyence (Osaka, Japan) VHX-7000, at a magnification of 200. Image stitches of 5 × 5 images were made to fully analyse the morphology of the tested samples. The arithmetic average surface roughness (*R_a_*) was measured on the 3D microscopy images, utilizing the short (λ_s_) and long (λ_c_) cut-off filters of 2.5 µm and 0.8 mm, respectively. The line roughness of each sample was measured at least 3 times, perpendicular to the abrasive-wear grooves, and 2–7 sampling lengths were available for every measurement.

The matrix–filler interface was investigated via scanning electron microscopy (SEM) images performed on a Phenom Pro (Thermofisher, Waltham, MA, USA) electron microscope. Produced samples were first submerged in liquid nitrogen for at least 2 min, after which they were cryogenically fractured. The fracture surfaces were then further investigated through SEM imaging. Cryogenic fractures are utilized so as to reduce the possibility of changing the surface morphology, due to the imposed shear during fracturing.

Melt flow index (MFI) measurements were performed on the virgin materials on a Tinius Olsen MP1200 (Horsham, PA, USA) machine with a nominal load of 2.16 kg at 200 °C for Rilsan BMNO (PA11) and 230 °C for Arnitel EL740 (TPC-E). The melt density, melt flow rate (MFR) and melt volume flow rate (MVR) values were recorded, together with the melt density, according to the standard ISO 1133.

Density measurements were performed according to ISO 1183-1, following the Archimedes method, on a Precisa (Dietikon, Switzerland) laboratory balance 205SM-DR. The liquid used for the measurements was ethanol, with a measured density of 0.795 g∙cm^−3^. The density of at least 8 different specimens of every compound was determined, after which the average and standard deviation were calculated.

Dielectric properties were measured by placing a sample with a thickness of 3 mm on top of a known substrate (RO4003C) (Advanced Connectivity Solutions, Chandler, AZ, USA) with copper coplanar waveguides (CPW). This allowed us to measure the influence of the material on the propagation properties of the electromagnetic wave through CPW. The dielectric constant was calculated by de-embedding the known substrate from the combined measurement. The measurements were performed with a Keysight (Santa Rosa, CA, USA) PNA-X over a frequency range from 10 MHz to 25 GHz, while only results up to 10 GHz are reported in the current work.

In order to find the dielectric constant of the polymer from the aggregated dielectric constant containing the influence of air, substrate and polymer, the filling factor (*FF*) needed to be determined. This *FF* defines the weighted average of the influence of all the materials on the aggregated dielectric constant. The filling factor for a CPW, GCPW or microstrip structure with an effective dielectric constant *ɛ_eff_* and physically composed of a substrate with dielectric constant *ɛ_sub_* and further surrounded by air (*ɛ* = 1), is given by

(1) $$FF=\frac{\varepsilon_{eff}-1}{\varepsilon_{sub}-1}$$

Thermal conductivity (TC) was measured on a Hot Disk TPS 2500S (Hot Disk, Göteborg, Sweden). All measurements were repeated 6 times at different locations of samples, with a sample thickness of 3 mm. The TC of the virgin polymer materials was measured according to the isotropic module, while the materials compounded with EG were measured according to the anisotropic module, following the ISO 22007 standard. A Hot Disk 7577 sensor (Hot Disk, Götenborg, Sweden) with Teflon insulation was used for all measurements. All samples were conditioned in a room at 23 °C ± 1 °C and 50 ± 10% relative humidity for at least 48 h prior to testing. Samples were tested under the same conditions after they had been conditioned.

Tensile tests were performed according to the ISO 527-1, on an Instron 5565 (Norwoord, MA, USA), with specimen type 1A, and the software program Bluehill 2 was used to analyse the data. An extension rate of 1 mm∙min^−1^ was used up to an elongation of 0.40% for determination of the tensile modulus between 0.05 and 0.25% extension, after which the secondary extension strain rate was 10 mm∙min^−1^ until failure of the sample. An Instron extensometer was used up until an elongation of 1.4% was attained.

Flexural tests were performed according to the ISO 178 on an Instron (Norwood, MA, USA) 4464 machine, with a strain rate of 2 mm∙min^−1^. The end-of-test strain rate was set to 15 mm. Analysis of flexural tests was carried out using the same Bluehill 2 software seen with the tensile tests.

Tribological tests were performed on a Wazau (Berlin, Germany) TRM 1000 pin-on-disc tribometer, with a normal load of 46 N and a sliding speed of 1 m∙s^−1^, which corresponds to a constant pv value (pressure multiplied by velocity value) of 0.92 (MPa∙m)∙s^−1^. The total sliding distance considered was 3000 m. Raw data were processed by means of a moving average, with a window size of 0.333% for the data points. The coefficient of friction (COF) was taken at a sliding distance of 2500 m, as every sample showed a steady state at this point. Counter surfaces were produced out of C45U (1.1730) tool steel, with an arithmetic average roughness (*R_a_*) value of 1.8 ± 0.1 µm, and maximal height of profile (*R_z1max_*) and total height of profile (*R_t_*) values of 13.7 ± 1.0 and 14.5 ± 1.0 µm, respectively. All samples were produced via injection moulding and had a diameter of 8 mm and height of 8 mm. The mass of the samples was recorded prior to and after testing in a conditioned state (23 °C ± 1 °C and 50 ± 10% relative humidity), so as to calculate the specific wear rate (*K*), according to Equation (2):

(2) $$K=\frac{∆V}{W\cdot L}=\frac{∆m}{W\cdot L\cdot \rho }$$

in which Δ*V* is volume loss, Δ*m* is the mass loss, *ρ* is the density, *W* is the applied normal load and *L* is the sliding distance.

## 3. Results and Discussion

### 3.1. Molecular Properties

To ensure the optimal distribution of the filler in the matrix, we first studied the thermal (DSC, TGA) and morphological (SEM) properties. It should be noted that such optimal distribution forms the foundation for the processing of the composite material and the realization of enhanced material properties, e.g., TC or resistance to wear.

**Figure 1:**
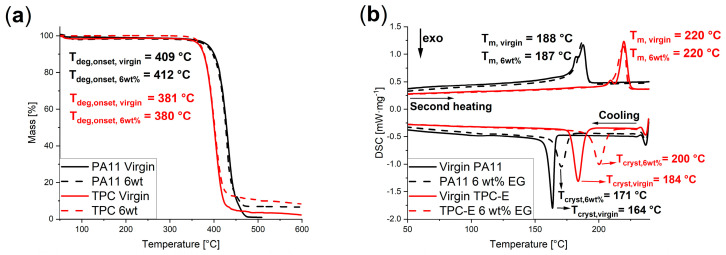
(**a**) TGA measurement curves for virgin (full lines) and 6 wt% (dashed lines) compounds with PA11 (black) and TPC-E (red) as a matrix material. (**b**) Cooling and second heating curves of DSC measurement for the virgin (full lines) and 6 wt% EG (dashed lines) compounds with PA11 (black) and TPC-E (red) as a matrix material.

Figure 1a displays the TGA measurement results for the virgin polymer matrix materials (full lines) and the compounds with 6 wt% of EG (dashed lines). For both PA11 (black) and TPC-E (red), no significant change in the degradation onset temperature (*T_deg,onset_*) is visible. The values for the virgin matrix material were 409 °C and 381 °C, respectively, while the degradation onset temperatures where EG was added was measured as 412 °C for the PA11 compound and 380 °C for the compound with TPC-E as a matrix material. The TGA tests were terminated after degradation of the polymer matrix, resulting in only the remaining EG as the final mass. The final masses recorded during TGA measurements showed wt% EG of 6.9% for PA11 and 5.3% for TPC-E, thus confirming that the composites produced had a correct loading level of EG, taking into account reasonable experimental error.

Similarly to the TGA results, the DSC results, as seen in Figure 1b, do not show significant changes in the melting temperature (*T_m_*). The crystallization peak temperature (*T_cryst_*), on the other hand, shows a slight shift to a higher temperature with EG added. This could be expected, as heterogenous crystallization will occur more easily, compared to homogeneous crystallization in a virgin polymer. This indicates that the added EG will serve as crystallization surfaces, for heterogenous crystallization, within the polymer matrix. The melting enthalpy of each polymer matrix increased slightly due to the addition of 6 wt% EG. The melting enthalpy during the second heating run increased slightly (ca. 10%) for both matrix materials, due to the addition of 6 wt% EG; more specifically, from 36.14 J·g^−1^ to 40.10 J·g^−1^ for PA11 and 34.10 J·g^−1^ to 37.33 J·g^−1^ for TPC-E, indicating that there is a small increase in the percentage of crystallinity. The latter result can again be linked to EG facilitating crystallization, as heterogenous crystallization will occur at a higher temperature as the number of crystal lattices increases.

Overall, it follows that the processability of the produced compounds will be similar to the processability of the virgin matrix materials, as the onset degradation temperature and the melting temperature do not change significantly.

**Figure 2:**
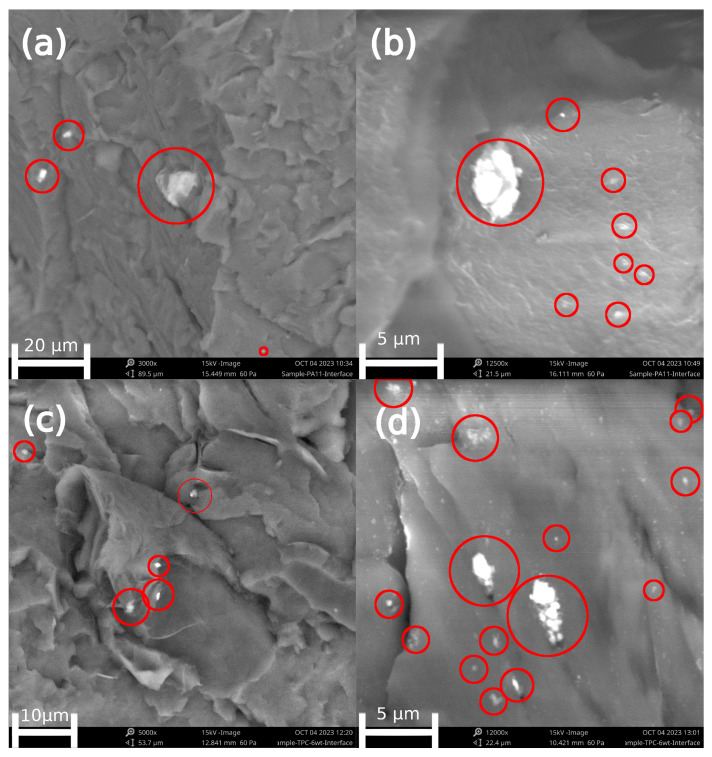
SEM Microscopy images of the cryogenically broken surfaces of PA11 with a magnification of (**a**) 3000 and (**b**) 12,500, and TPC-E matrix with magnification (**c**) 5000 and (**d**) 12,000.

The SEM images are included in Figure 2 and relate to imaging for cryogenically broken samples to evaluate the morphology and matrix-filler interactions. In more detail, Figure 2a,b show images of the PA11 composite with 6 wt% of EG. In both images, no defined interface can be observed between EG and matrix, indicating efficient matrix–filler interactions. Figure 2c,d show, in turn, images of the TPC-E composite with 6 wt% of EG. Similarly to the images for PA11, it can be seen that the matrix materials seem to embed EG very nicely. Furthermore, no voids can be seen for any of the matrix materials used. Some agglomerations of fillers, however, are visible in Figure 2, indicating that the dispersion of filler was not optimal, which will influence the change in properties as well, as stated by Wieme et al. [2]. It should be noticed that the dispersion of the filler is influenced not only by interactions with the matrix, which are favourable in our case, but also by the shear during processing.

From Figure 2, it is further visible that no EG particles are found with a perpendicular orientation to the other particles. This shows that there was a high influence of the processing method on the final orientation of the EG particles within the polymer matrix and all (or as good as all) the EG particles are aligned parallel to the flow pattern. Linking this to the work of Wieme et al. [2], this indicates a non-uniform TC control.

**Figure 3:**
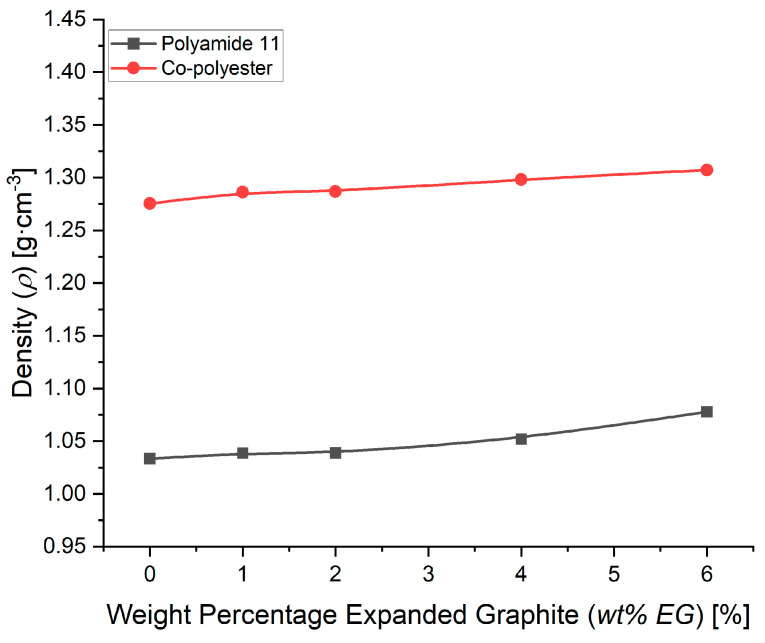
Specific density as measured for the compounds with increasing wt% EG and matrix materials PA11 (black) and TPC-E (red). B-spline trendlines were added to indicate the trend taken from the current measurement results.

Figure 3 shows the specific density measurement results for the composites with increasing weight percentage (wt%) EG. An increasing trend in specific density with increasing wt% EG is observed using both matrices. This seems to contradict the EG datasheet, but it should be remembered that this datasheet states the bulk density of the powder. The density of the EG powder stated is expected to be much lower than the specific density of the powder itself. The overall higher specific density of the TPC-E material is as expected, as the specific density stated in the datasheet of the virgin TPC-E has a value of 1290 kg·m^−3^ compared to a value of 1030 kg·m^−3^ for the virgin PA11 material.

### 3.2. Thermal Conductivity and Dielectric Characterisation in View of Microelectronic Applications

**Figure 4:**
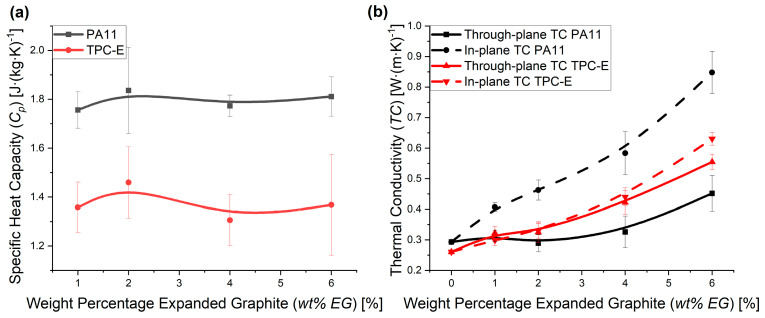
(**a**) Specific heat capacity (*C_p_*) results from PA11 (black) and TPC-E (red). (**b**) Through-plane (solid lines) and in-plane (dashed lines) thermal conductivity (TC) results for PA11 (black) and TPC-E (red). B-spline trendlines were added to indicate the trend taken from the current measurement results.

As the EG fillers are oriented due to the manufacturing process [2,13], as was observed via SEM (Figure 2), thermal conductivity measurements were performed in several directions to evaluate the thermal properties of the composites. Prior to the TC measurements, the specific heat capacity (*C_p_*) values were measured. Figure 4a shows that no difference in *C_p_* can be noticed, due to the addition of EG. Furthermore, the *C_p_*-value of PA11 is generally higher than the value for TPC-E, highlighting a strong difference and noting the lower specific densities in Figure 3 for PA11.

In Figure 4b, both the through-plane (solid line) and in-plane (dashed line) TC measurement results are presented and compared to the state-of-the art relatively high TC values found at 6 wt% EG. While the measurement results for PA11 (black) show a significant difference in through-plane and in-plane TC values, the difference is not so pronounced for the TPC-E (red) matrix composites. In the latter case, a difference between both values can only be noticed for the 6 wt% EG samples, highlighting the relevance of this amount. The difference in through-plane and in-plane TC can be explained due to the processing method [2]. As explained above, due to the injection moulding process used to produce the samples, the orientation of most EG particles is aligned in the direction of the material melt flow, which is along the part [2], as we also observed via SEM (Figure 2). This leads to a more optimal thermal conductive network being formed in-plane, compared to the perpendicular direction.

The difference between both composites for the relative importance of both TC values can be related to basic rheological analysis. The MFR results for both materials are not significantly different, as 19.7 ± 2.4 g∙(10 min)^−1^ and 21.6 ± 1.5 g∙(10 min)^−1^ were the results for TPC-E and PA11, respectively. Melt volume flow-rate (MVR) results, however, did indicate that PA11 is significantly less viscous, as MVR values of 17.8 ± 1.6 cm^3^∙(10 min)^−1^ and 24.0 ± 0.7 cm^3^∙(10 min)^−1^ were found for TPC-E and PA11, respectively. This is related to a lower melt density for the virgin PA11 material (0.9 ± 0.1 g∙cm^−3^) compared to TPC-E (1.1 ± 0.1 g∙cm^−3^). Consequently, during processing, PA11 flows more easily, creating more easily the orientation of the filler. The latter enhances the in-plane TC for the PA11-based composite.

Intriguingly, no clear PT is visible in Figure 4b, indicating that this threshold has not yet been reached with 6 wt% of EG. Li et al. [49] showed in their study that a further increase in TC can be attained with a much larger wt% of EG, while Sever et al. [36] showed that the mechanical properties showed even more significant change up to a loading level of 40 wt% EG. The current study aims to keep the processability and mechanical properties of the composite materials similar to those of the virgin polymer matrix materials. Low loading levels of EG (up to 6 wt%) were therefore used, as the research by Sever et al. [36] indicated that mechanical properties changed significantly, starting from 10 wt%.

**Figure 5:**
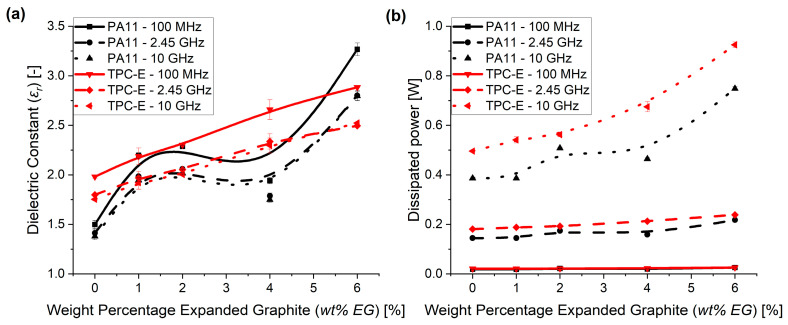
(**a**) Dielectric constant measurement results for the frequencies 100 MHz (solid line), 2.45 GHz (dashed line) and 10 GHz (dotted line) for both PA11 (black) and TPC-E (red) material. (**b**) Dissipated power as measured for PA11 (black) and TPC-E (red) at frequencies 100 MHz (solid line), 2.45 GHz (dashed line) and 10 GHz (dotted line). B-spline trendlines were added to indicate the trend taken from the current measurement results.

Similar to the TC data, an increasing trend can be noticed for the dielectric constant (*ɛ_r_*) measurements, as seen in Figure 5a, in cases where the loading level of EG increases. Furthermore, similar to the study of Xie et al. [48], a decrease in *ɛ_r_* is visible with increasing frequency. The frequencies in the study of Xie et al. were, however, limited to the range of 1 to 10^6^ Hz, which explains why much higher dielectric constants (e.g., as high as 180 at 50 Hz) were achieved within their research. Goyal et al. [34] stated that the interfacial dipoles do not have enough time to orient themselves at higher frequencies, which explains the large differences in dielectric constant between previously reported values for low frequencies and the values achieved in the current research. The decrease in dielectric constant with increasing frequency was also noted by Lecoublet et al. [50], and was allocated to the limitation of dipole mobility as the frequency increased.

In Figure 5b, the dissipated power is presented as a function of the filler content amount. Only a small increase with increasing loading levels of EG at lower frequencies, i.e., 100 MHz (solid line) and 2.45 GHz (dashed line), is observed. In contrast, the dissipated power at a frequency of 10 GHz (dotted line) nearly doubled upon comparing the virgin polymer and the compounds with 6 wt% EG. This makes these 6%-based composites promising materials for EMI shielding applications [51], as 8.3–12.4 GHz is commonly used within communication applications [52]. The large rise in EMI shielding effectiveness was also noticed in the study of Sankaran et al. [53], in which 7 wt% of graphene was added within a PVDF matrix.

Overall, it follows that no clear PT can be noticed for the TC and that the dielectric values are rather low for low EG loadings, but still an increase in both properties resulted. The increase in dissipated power especially, obtained for very high frequencies (i.e., 10 GHz), is very interesting. The dissipated power is expected, however, to lead to a high increase in temperature of the polymer matrix and, as the TC did not yet show a PT, the increase in dissipated power could lead to a non-acceptable increase in temperature of the polymer material. Hence, it could be that a slight increase in the EG amount is needed for use at higher frequencies.

### 3.3. Mechanical and Tribological Characterisation in View of Durabillity and Wear Resistance

**Figure 6:**
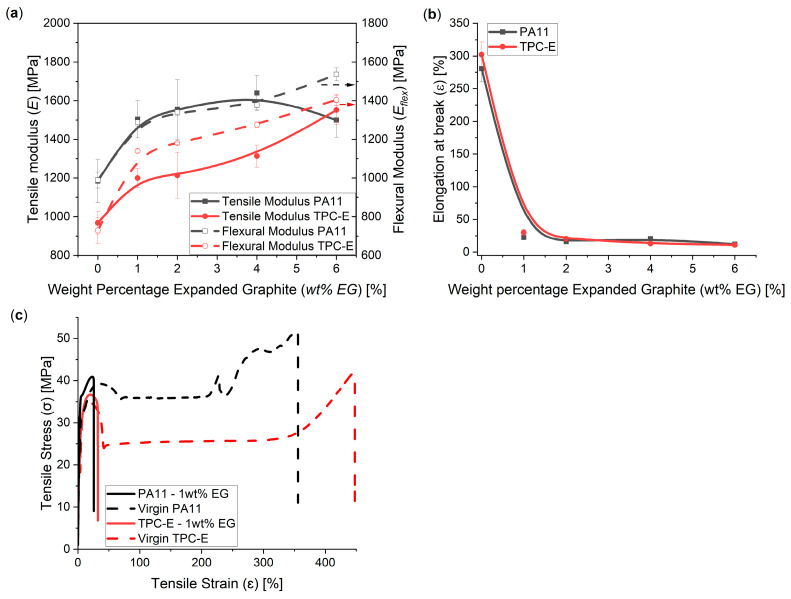
(**a**) Tensile modulus (full lines) and flexural modulus (dashed lines) of compounds with increasing wt% EG, using PA11 (black) and TPC-E (red) as a matrix material. (**b**) Tensile elongation at break, as measured for compounds with increasing wt% EG, using PA11 (black) and TPC-E (red) as a matrix material. (**c**) Example tensile test curves of the virgin material (dashed line) and the 1 wt% EG compounds (full line), for matrix materials PA11 (black) and TPC-E (red). B-spline trendlines were added to indicate the trend taken from the current measurement results.

The results for tensile and flexural modulus measurements can be seen in Figure 6a. Both the tensile modulus (*E*) (full line) and flexural modulus (*E_flex_*) (dashed line) show a significant increase after the initial introduction of EG (1 wt%), which is similar to the effect observed by Potts et al. [54]. The increasing trend in elastic modulus (*E*) (Figure 6a) is, however, opposite to the trend in the research by Difallah et al. [42] with graphite powder, but, in any case, the decreasing trend for the elongation at break (Figure 6b) is similar; Difallah et al. [34] noted a drop in elongation at break starting at the initial introduction of graphite powder in an acrylonitrile butadiene styrene (ABS) matrix.

An increase in EG loading levels leads to a general increasing trend in the modulus values, making the material stiffer. This is also supported by the tensile test graphs in Figure 6c and the elongation-at-break results in Figure 6b. This differs slightly from the results found by Zheng et al. [23], as they found in their study that the modulus only increased slightly, due to a poor matrix–filler interface for the HDPE-EG composite. In that study, the increase in modulus, despite the fact that a poor matrix–filler interface was present, was allocated by the authors to the higher tensile properties of the expanded graphite. However, in the current study, a more significant increase in tensile modulus was found due to the high stiffness of the EG and a good matrix–filler interface (Figure 2).

A more detailed analysis in Figure 6 reveals that the virgin polymer materials (dashed line) show a very ductile behaviour if a tensile load is applied. The elongation at break for virgin matrix materials, as can be seen in Figure 6b, was 281 ± 20% for virgin PA11 and 302 ± 19 for virgin TPC-E. These values decreased to 22 ± 3% and 30 ± 5%, respectively, at the initial introduction of 1 wt% of EG. It further follows from Figure 6c that the virgin polymer materials showed strain hardening near the end of the tensile tests, with PA11 also showing some signs of necking. The composites, on the other hand, did not show this ductile behaviour, as fillers within the polymer matrix acted as stress concentrators, therefore raising the localized stresses before failure occurred [20]. The introduction of filler thus clearly induces a ductile–brittle transition. Similarly, Zheng et al. [20] reported an increase in tensile modulus and a significant decrease in elongation at break where HDPE was reinforced with 3 wt% of EG. Furthermore, the results are in line with the results found by Liu et al. [55], as their results indicated a strong improvement in elastic modulus through the addition of 1 wt% of GO in a polyimide matrix.

**Figure 7:**
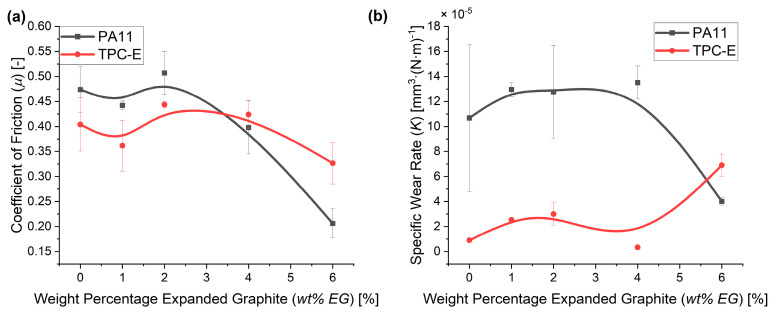
(**a**) Coefficient of friction (COF) at sliding distance 2500 m for PA11 (black) and TPC-E (red) matrix with increasing loading levels of EG. (**b**) Specific wear rate (*K*), as measured for PA11 (black) and TPC-E (red) matrix, with increasing loading levels of EG. B-spline trendlines were added to indicate the trend taken from the current measurement results.

Figure 7a shows the coefficient of friction (COF) as measured during pin-on-disc tests. Statistical analysis (one-sided *t*-test) showed that for the COF of PA11 significant differences could only be noted starting from 4 wt% of EG, while the decrease in COF is only significant at 6 wt% in the case of the TPC-E. For PA11, the COF decreased from 0.47 to 0.21 between the virgin polymer material and the 6 wt% composite, while the COF for TPC-E material was 0.40 for the virgin polymer material and 0.33 for the 6 wt% EG composite material. Via the introduction of EG, we were thus able to improve the tribological properties of the composites. These results are in line with the results of the study by Sathees et al. [41], noting a reduction in COF for polyamide 6 (PA6) composites with a loading level of 5 wt% of graphite. The observed reduction in their study continued with increasing percentages of graphite (up to 40 wt%).

**Figure 8:**
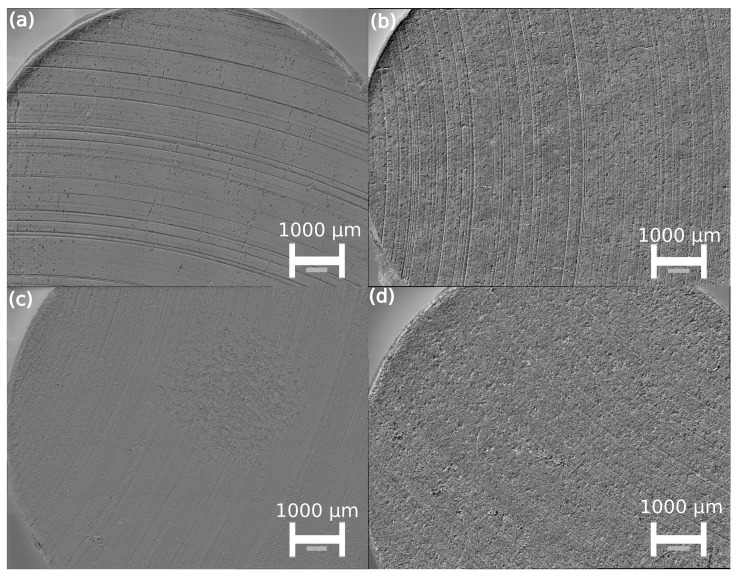
Microscopic images made of pin samples after pin-on-disc test at a magnification of 200 of (**a**) virgin PA11—mainly abrasive wear; (**b**) PA11—6 wt% EG—much higher amount of adhesive wear, with signs of abrasive wear; (**c**) Virgin TPC-E—mainly abrasive wear, with signs of adhesive wear; (**d**) TPC-E—6 wt%—much higher amount of adhesive wear, with signs of abrasive wear.

Figure 7b indicates that the specific wear rate (*K*), as calculated via Equation (2), reduces for PA11 once the COF reduces, while for TPC-E *K* slightly increases. It should be noted that during the measurements the end-of-test contact temperature was between 80 and 120 °C and that on the countersurface a transfer layer was formed. This is also supported by the microscopic images of the tested samples, as seen in Figure 8. Figure 8a,c show tribological samples of the virgin PA11 and virgin TPC-E, respectively, whereas Figure 8b and Figure 8d show the samples of the composites with 6 wt% EG, with matrix materials PA11 and TPC-E, respectively.

From Figure 8a it can be seen that the main wear mechanism for the virgin PA11 material is abrasive wear [56]. This can be seen, as the grooves visible are created by ploughing and micro-cutting, and by the asperities of the rough C45U countersurfaces during testing. Furthermore, small traces of adhesive wear are visible on the tested surface of the counter surfaces. Despite the fact that Figure 8c shows very similar results for the virgin TPC-E material, fewer deep cuts can be seen and a larger number of traces produced by adhesive wear are visible. This is supported by the COF results in Figure 7a, as a smaller COF resulted after 2500 m of sliding. This leads to the conclusion that a better and more significant self-lubricating transfer layer could be formed by the virgin TPC-E material, compared to the virgin PA11 material.

Upon focusing only on the 6 wt% EG composites, as visible in Figure 8b,d, the traces of abrasive wear are still present, although to a smaller extent. Furthermore, it can be noticed for both compounds that the morphology of the samples indicates a larger amount of adhesive wear. Combining the latter results with the results from Figure 7a allows to postulate that the addition of EG increased the amount of adhesive wear and improved the creation of a transfer layer and that the anti-frictional properties of EG led to a decrease in COF for both matrix materials. The transfer layer has been found to have self-lubricating [46] effects, leading to a lower COF, which could be even more significant in cases where a much larger wt% of EG is added and can be of interest for the continued development of triboelectric generators [40].

*R_a_* values, as measured for the virgin samples after pin-on-disc tests, were found to be 1.5 ± 0.2 µm for both PA11 and TPC. Measurements for the 6 wt% composite materials showed *R_a_* values of 1.0 ± 0.1 µm and 1.2 ± 0.2 µm for PA11 and TPC composites, respectively. These values indicate that adhesive wear results in a more uniform wear of the complete pin-sample surface, compared to the wear present as a result of abrasive wear.

## 4. Conclusions

In this study, polyamide 11 (PA11) and thermoplastic co-polyester (TPC-E) were filled with low weight percentages (wt%) of expanded graphite (EG) via melt mixing, aiming at the manufacturing of a composite material with enhanced thermal and electric conductivity while maintaining similar settings from the virgin processing and controlling the mechanical and tribological properties.

It was shown that the thermal conductive and dielectric properties of both polymer matrices can be enhanced with low weight percentages of EG, while keeping the processability similar to the virgin polymer matrix processability. This was carried out via the most industry-relevant processing technique, allowing the development of new use cases with polymer composite materials, combining beneficial properties of the polymer matrix, e.g., flexibility, and the enhanced properties obtained through low loading levels of EG fillers.

Thermal analysis via TGA and DSC measurements showed no differences in onset degradation temperature and melting temperature, while the crystallization temperature shifted to slightly higher (ca. 6 °C for PA11 and 16 °C for TPC-E) temperatures. Slightly higher percentages (ca. 10%) of melting enthalpy were found, as the addition EG within the matrix materials allowed for heterogeneous crystallization, also explaining the increase in crystallization temperature. Morphological analysis of cryogenically broken samples showed a good matrix–filler interface and flow-induced orientation of the filler.

Dielectric and thermal conductive properties were enhanced as well, through the addition of EG, although the percolation threshold could not be attained. A difference in in-plane and through-plane thermal conductivity was visible for both materials, albeit at different loading levels of EG. MFR measurements indicated that the les-viscous matrix material, i.e., PA11, showed a higher tendency to orient the filler particles during processing, explaining the difference in the in-plane and through-plane thermal conductive network that could be formed at 1 wt% EG. The dielectric constant remained rather low, compared to previous studies, due to the high frequencies that were considered. However, the dissipated power showed a much larger increase at high frequencies.

Furthermore, the tensile and flexural modulus showed an increase, starting from a very low filler content of EG. The elongation at break showed a significant decrease at the initial introduction of 1 wt% of EG within both matrix materials.

In addition, the coefficient of friction of the produced composite materials only showed a significant decrease at 4 and 6 wt% EG, for PA11 and TPC-E, respectively. The specific wear rate, on the other hand, showed a reduction at 6 wt% for the PA11-composites, while the specific wear rate of TPC composites showed a significant increase. Morphological investigation of the tested surfaces showed that the main wear mechanism changed from abrasive to adhesive wear. The decrease in coefficient of friction and the change in main wear mechanism were attributed to the addition of EG, as EG promotes the creation of a transfer layer and is known to be a solid lubricant.

Hence, to optimize all properties studied, one needs to compromise by either slightly lowering the EG content versus the 6% loading case or increase this content in cases where TC strong increases need to be achieved.

## Figures and Tables

**Table 1:** Processing conditions for compounds with matrix materials: (left) Rilsan BMNO, Arkema (PA11) and (right) Arnitel EL 740, DSM (TPC-E).

	Rilsan BMNO (PA11)	Arnitel EL 740 (TPC-E)
Temperature Profile [°C]	240—240—225—210	245—230—215—205
Mold Temperature [°C]	40	50
Injection Speed [mm·s^−1^]	60	60
Clamping force [kN]	800	800
Holding Pressure [bar]	24	52
Dosing Speed [%]	70	60
Dosing pressure [bar]	10	4

## Data Availability

The raw data are available upon reasonable request to the corresponding author.
